# Atomic Scale Simulation on the Fracture Mechanism of Black Phosphorus Monolayer under Indentation

**DOI:** 10.3390/nano8090682

**Published:** 2018-09-01

**Authors:** Yang Liu, Yuhong Liu, Jianbin Luo

**Affiliations:** State Key Laboratory of Tribology, Tsinghua University, Beijing 100084, China; liuyang2013@gmail.com

**Keywords:** molecular dynamics simulation, black phosphorus, indentation, deformation, rupture

## Abstract

Molecular dynamics simulations on the indentation process of freestanding and Pt(111)-supported black phosphorus (BP) monolayer were conducted to study the fracture mechanism of the membrane. For the freestanding BP monolayer, crack grows firstly along armchair direction and then zigzag direction during the indentation process. Whereas, for the Pt(111)-supported BP monolayer, crack growth shows no obvious directionality, with irregular distribution of crack tips. Further study on stress distribution shows that maximum normal stress component at elastic stage is in zigzag direction for the freestanding BP monolayer, and in vertical direction for the Pt(111)-supported BP monolayer. As BP monolayer is remarkably anisotropic for in-plane mechanical properties and homogeneous for out-of-plane mechanical properties, the difference of stress state may be a key reason for the different fracture behavior in these two cases. These findings may help to understand the failure mechanism of BP, when applied in nano-devices.

## 1. Introduction

Two dimensional (2D) materials, such as graphene, few layer MoS_2_, and black phosphorus (BP), have showed great application potential in many domains due to their unique physicochemical properties [1,2,3]. For instance, the fore-mentioned three materials can be used as lubricating films [4,5,6] and protective coatings [7], taking advantage of their atomic scale thickness [8], excellent mechanical properties [9,10], and good thermal conductivity [11]. Due to the large band gap, boron nitride (BN) can be used as gate insulation layer in micro and nanoeletronic devices [12]. Moreover, the unique mechanical and electrical properties of BP film make it a promising material in strain-dependent optoelectronics, flexible electronics, and nano- or micro-electromechanical devices [13]. Due to the high hole mobility and on-off ratio of photo-induced current, BP can be used to make room-temperature terahertz (THz) photodetectors [14]. However, most applications of these 2D materials rest on their structural integrity [15]. For example, when used as lubricating film or protective coatings on rubbing surfaces, the friction reduction and wear protection effect will vanish once the 2D layers rupture [16].

Studying the deformation and fracture properties of materials is important for avoiding mechanical failures in applications [17]. Fracture mechanism of 2D material is very different from bulk materials, as 2D materials are more sensitive to defect [18,19], structural changes [20], bond breakage and crack tips with atomic sharpness [21,22]. So far, studies on the fracture mechanism of 2D materials are scarce, and most of these studies are limited to graphene and MoS_2_. Structure of BP monolayer differs greatly from the truly planar structure of graphene and the sandwich structure of MoS_2_. The unique corrugated structure formed by two P atom layers in BP monolayer can lead to unique mechanical behaviors and properties [23]. For instance, both experiments and molecular simulations have shown that the mechanical properties of BP layer are highly anisotropic [24,25], as the elastic modulus and rupture strength in zigzag direction can be two times larger than armchair direction [26]. These unusual properties of BP imply a different fracture process and mechanisms. Nano-indentation is a common approach to calculate the mechanical properties of crystalline materials, and can be used to study the fracture behavior of layered materials both in experiments and molecular simulations. For example, using a nano-indentation device, fracture toughness of single-crystal Bi_2_Te_3_ can be measured [27]. 

In this work, molecular dynamics simulations on the indentation process of a freestanding and a Pt(111)-supported BP monolayer were conducted. Crack formation and propagation were observed. Stress distribution at different indentation stage, including normal stress and von Mises stress, were studied. The fracture mechanism of BP monolayer was then illustrated.

## 2. Simulation Details 

**Figure 1:**
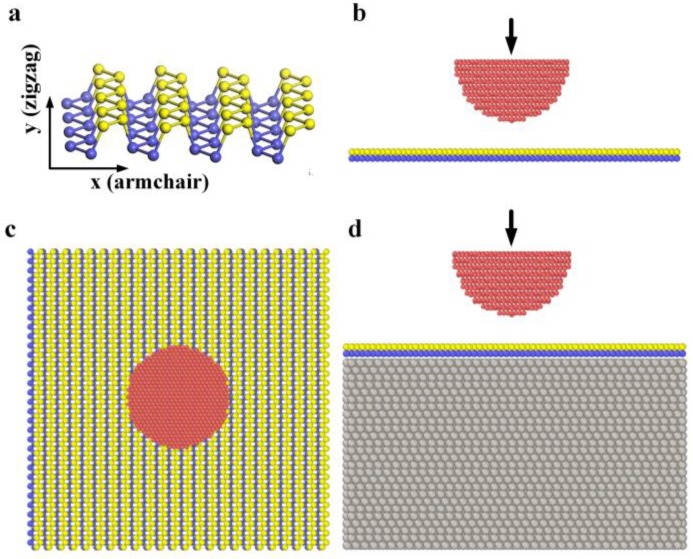
(**a**) Atomic structure of the black phosphorus (BP) monolayer. P atoms at the top layer are shown in yellow while P atoms at the bottom layer are shown in blue; (**b**,**c**) Left and top view of the simulation model for the indentation process of a freestanding BP monolayer; (**d**) Left view of the simulation model for the indentation process of a Pt(111)-supported BP monolayer.

A hemisphere diamond tip with a radius of 18 Å cutout from a (111)-oriented diamond crystal was placed right above the freestanding or Pt(111)-supported BP monolayer, as shown in Figure 1. The tip was set rigid in order to avoid the influence of dangling bonds on its surface. The initial distance between the diamond tip and BP monolayer is 10 Å. The size of BP monolayer is 105.92 × 105.12 Å^2^. Pt is a common substrate to grow graphene and MoS_2_ on its surface [16,28]. In this work, the Pt(111) substrate has a thickness of 59.2 Å, and a same horizontal size with BP monolayer. In *x* and *y* direction, periodic boundary conditions were used. In *z* direction, the simulation box was non-periodic and shrink-wrapped with a minimum value. During the indentation process, the diamond tip moved vertically to the BP monolayer, at a speed of 1.0 m/s. The indentation process of freestanding monolayer at different loading speed was also simulated, and the results can be found in supplementary material. When indentation speed of the tip is less than 10 m/s, indentation speed has little influence on the results of indentation process.

An embedded atom method (EAM) potential was used to simulate the interaction between Pt atoms [29]. Stilling Webber potential parameterized by Jin-Wu Jiang [30,31,32] was used to simulate the interaction between P atoms in the BP monolayer. Tersoff potential was used to describe the interaction between carbon atoms in the diamond tip [33]. The covalent interactions between the diamond tip and the monolayer were neglected, and a C-P Lennard Jones (LJ) potential was used to model the Van der Waals forces between the diamond tip and BP monolayer. Similarly, a C-Pt LJ potential was used to model the Van der Waals forces between the diamond tip and Pt(111) substrate, and a P-Pt LJ potential was used to model the Van der Waals forces between BP monolayer and Pt(111) substrate. Parameters for LJ potential are shown in Table 1. Here, in this work, parameters for C-P LJ potential were taken from Reference [34], and parameters for C-Pt and Pt-P were obtained by Lorentz-Berthelot mixing rules [35], with the original ε and σ parameters for C-C, Pt-Pt, P-P taken from References [34,36,37]. Distance between theBP monolayer and Pt(111) substrate is 2.91 Å, which is the equilibrium distance of these two materials. The cut off radius of LJ potential is set to 10 Å. High temperature can arouse the fluctuation of atom distances, and induce the fluctuation of atom stress. In order to avoid the influence of temperature on atom stress and focus on the mechanical effect of the indentation process, Langevin thermostat was adopted to control a constant temperature of 0.01 K during the simulations [38]. The extremely low temperature may arouse more gas adsorption of black phosphorus in practical applications [39], and researchers have tried to minimize surface chemical reactivity of black phosphorus [40], which may extend the applications of black phosphorus. All the simulations were carried out with LAMMPS (version 9 December 2014) [41].

## 3. Results and Discussions

### 3.1. Indentation Process and Fracture Behavior

**Figure 2:**
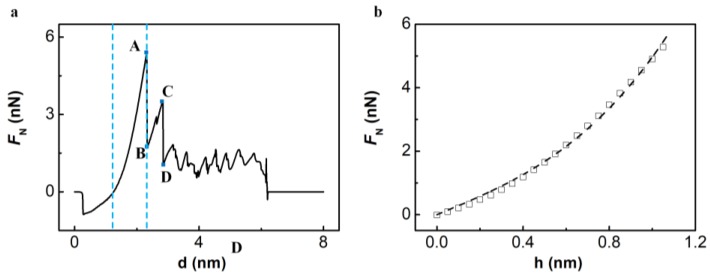
(**a**) The force-displacement (F_N_(d)) curve for the indentation process of the freestanding BP monolayer; (**b**) The force displacement curve at elastic stage (corresponding to curve between the two blue dashed line in Figure 2a), and the dashed line is a nonlinear fit of Formula (1).

During the indentation process, the freestanding BP monolayer will go through two different stages: An elastic deformation stage, and a plastic deformation stage, as shown in Figure 2a. During the elastic stage, the force displacement curve is smooth and monotonically increasing (see curve on the left side of point A in Figure 2a). During the plastic deformation stage, the force displacement curve is rough with sudden drops and sawtooth shaped steps (see curve on the right side of point A in Figure 2a).

Fracture of the freestanding BP monolayer is firstly brittle-like and then ductile-like. During the indentation process of the freestanding BP monolayer, there are two instantaneous drops in F_N_ (see Figure 2a, point A→B, C→D). After the two cracks in BP monolayer, the fracture behavior is ductile-like, with sawtooth shaped steps of F_N_(d) curve (curve on the right side of point D in Figure 2a). After complete rupture of the freestanding monolayer, interaction force between the tip and monolayer will drop to zero in vertical direction, as shown in Figure 2a.

Indentation of 2D materials is a common approach to measure their elastic modulus and rupture strength [42]. With BP monolayer regarded as a linear isotropic elastic material, the indentation process of the freestanding BP monolayer can be approximated as central point loading on a clamped circular membrane [43]. The relationship between indentation force and displacement at elastic stage can be deduced as Formula (1) shows [44]:

*F* = *σ*_0_^2D^π(*h* − *h*_0_) + *E*^2D^*q*^3^(*h* − *h*_0_)^3^/*a*^2^, (1)

wherein, *F* is the point load at the center of the membrane, *h* is vertical displacement of the tip, *h*_0_ = 1.0 nm is the initial distance between the diamond tip and BP monolayer, *h* − *h*_0_ can represent the deflection at the center point approximately, a is the radius of BP monolayer, *σ*_0_^2D^ and *E*^2D^ is the pretension and elastic modulus of the membrane, *q* = 1/(1.05 − 0.15*ν* − 0.16*ν*^2^) is a dimensionless constant, and *ν* = 0.4 is the Poisson ratio of BP in the perpendicular direction [45].

The elastic modulus of BP monolayer can be obtained through a nonlinear fit of the smooth curve (curve on the left side of point A) in Figure 2a to Formula (1), as shown in Figure 2b. The result of *E*^2D^ is 43.66 GPa, greater than *E*_armchair_ and less than *E*_zigzag_ obtained by first-principle calculations [46].

**Figure 3:**
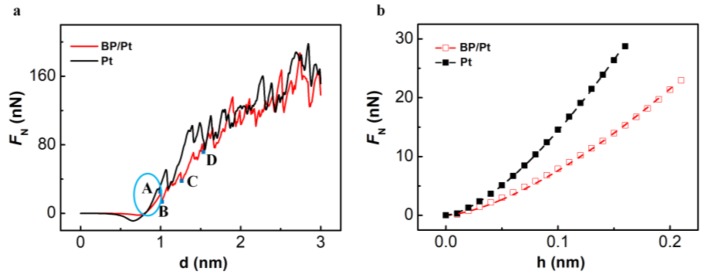
(**a**) The force-displacement (*F*_N_(*d*)) curve for the indentation process of Pt and BP/Pt substrate; (**b**) The force displacement curve at elastic stage (corresponding to the blue ellipse in Figure 3a), and the dashed line is a nonlinear fit of Formula (2).

There are also two different stages during the indentation process of Pt-supported BP monolayer: An elastic deformation stage and a plastic deformation stage, as shown in Figure 3a. Differently with the freestanding BP monolayer, the elastic deformation stage (blue ellipse area in Figure 3a) of Pt-supported BP monolayer is shorter.

In the elastic deformation stage of nano-indentation process, Hertz contact model can be used to evaluate the hardness of the substrate. For the Hertz contact of a rigid ball with an elastic plane, the relationship between load and the indentation depth can be expressed by Formula (2):

*F* = (4/3)(1 − *ν*_1_^2^)*R*^0.5^*h*^1.5^/*E* (2)

wherein, *F* is the interaction force in the vertical direction between the diamond tip and the substrate, *R* = 1.8 nm is the radius of the diamond tip, h is the indentation depth, *E* is the elastic modulus of the substrate, and *ν*_1_ is the Poisson’s ratio of the substrate. Here we take *ν*_1_ = 0.35 for Pt(111) substrate, and *ν*_1_ = 0.4 for BP-covered Pt(111) substrate.

Elastic modulus of Pt(111) and BP-covered Pt(111) substrate can be deduced by fitting the *F*_N_(*h*) curve at elastic stage to Formula (2), as shown in Figure 3b. It can be found that the simulation results are in good agreement with Hertz contact theory. Elastic modulus of Pt(111) in this work is 223.5 GPa, which is very close to results of theoretical calculations by Romasco [47]. Elastic modulus of BP-covered Pt(111) in this work is 112.9 GPa. 

The indentation hardness of the substrate can be defined as Formula (3):

*H* = *F*_max_/*A*_c_, (3)

wherein, *F*_max_ is the maximum indentation force at elastic stage, and *A*_c_ is the corresponding projected contact area. For the hemisphere indentation tip used in this work, the projected contact area can be calculated by Formula (4). *F*_max_ = 28.84 nN for Pt substrate and 22.95 for BP/Pt substrate.

*A*_c_ = π(2*Rh*_max_ − *h*_max_^2^) (4)

In Formula (4), *R* is the radius of the diamond tip and *h*_max_ is the maximum indentation force at elastic stage. *h*_max_ = 0.161 nm for Pt substrate and 0.211 nm for BP/Pt substrate. The indentation hardness of Pt substrate and BP/Pt substrate can be calculated by Formulas (3) and (4). The indentation hardness of Pt(111) substrate is 22.95 GPa, and the indentation hardness of BP/Pt substrate is 10.22 GPa.

Compared with bare Pt substrate, load for BP-covered Pt substrate is smaller before rupture as shown in Figure 3a, which indicates BP coating on Pt surface will not enhance the load bearing capacity of the substrate like graphene and MoS_2_ monolayer [16,48]. The maximum load BP/Pt substrate can bear at elastic stage is 52.82 nN, which far exceeds 5.45 nN for the freestanding BP monolayer, hence the stress state in these two cases can be very different. While crack formation and propagation in the freestanding BP monolayer complete in a very short time, the Pt(111)-supported BP monolayer undergoes a plastic deformation stage (see curves with sawtooth shaped steps from point A to D, in Figure 3a), before completely rupture during the indentation process, which means the fracture of the Pt(111)-supported BP monolayer is ductile-like. After the complete rupture of the Pt-supported BP monolayer, the force-displacement curve nearly overlapped with curve of bare Pt substrate, as shown in Figure 3a.

**Figure 4:**
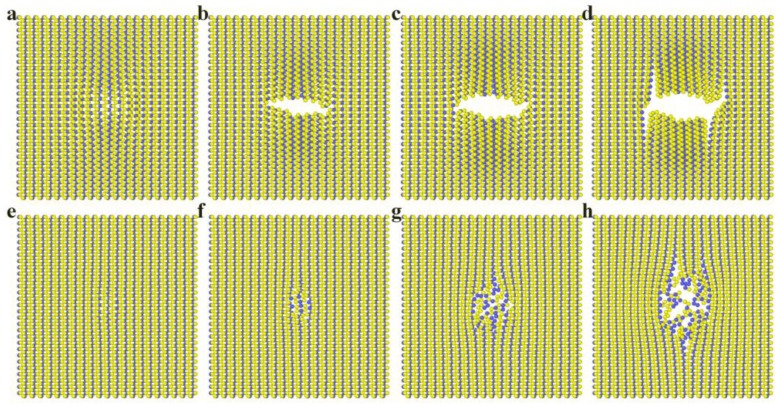
(**a**–**d**) Top view of the freestanding BP monolayer during the indentation process, and Figure a, b, c, d correspond to point A, B, C, D in Figure 2a respectively; (**e**–**h**) Top view of the Pt(111)-supported BP monolayer during the indentation process, Figure e, f, g, h correspond to point A, B, C, D in Figure 3a respectively.

Figure 4 shows the fracture behavior of the freestanding and Pt(111)-supported BP monolayers. Cracks in the freestanding BP monolayer are directional and occur in order, while cracks in the Pt(111)-supported BP monolayer are of no directionality and out of order. Crack in the armchair direction shown in Figure 4b corresponds to the sudden drop of force-displacement curve from point A to B in Figure 2a, and crack in the zigzag direction shown in Figure 4d corresponds to the sudden drop from point C to D in Figure 2a.

As elastic modulus in the zigzag direction can be two times higher than armchair direction [24], the mean stress in zigzag direction will also be higher than armchair direction at the same deflection of the freestanding BP monolayer. The higher stress in zigzag direction may be a reason why crack grows firstly in a direction perpendicular to zigzag direction (i.e., in the armchair direction). In order to further study the relationship between stress state and the fracture behavior, stress distributions at different indentation stage were investigated next.

### 3.2. Stress Distribution

Stress field is a key factor to identify the crack propagation behavior of materials. Here, Virial stress tensor, together with Voronoi volume calculated by LAMMPS were applied to calculate the atomic Virial stress [49] using Formula (5):

*σ*_ab_ = *S*_ab_/*V*_voronoi_ (5)

wherein, *a* and *b* take on value *x*, *y*, *z* to generate the six components of the symmetric atom-stress tensor, *V*_voronoi_ is the per-atom Voronoi volume calculated by VORONOI package of LAMMPS, and *S*_ab_ is the per-atom Virial stress tensor. The tensor form of the Virial stress can be converted into a scale value by the von Mises formulation for the yield criterion [50], as shown in Formula (6):

*σ*_s_ = √1/2*√[(*σ*_x_ − *σ*_y_)^2^ + (*σ*_y_ − *σ*_z_)^2^ + (*σ*_x_ − *σ*_z_)^2^ + 6(*τ*_xy_^2^ + *τ*_yz_^2^ + *τ*_zx_^2^)] (6)

**Figure 5:**
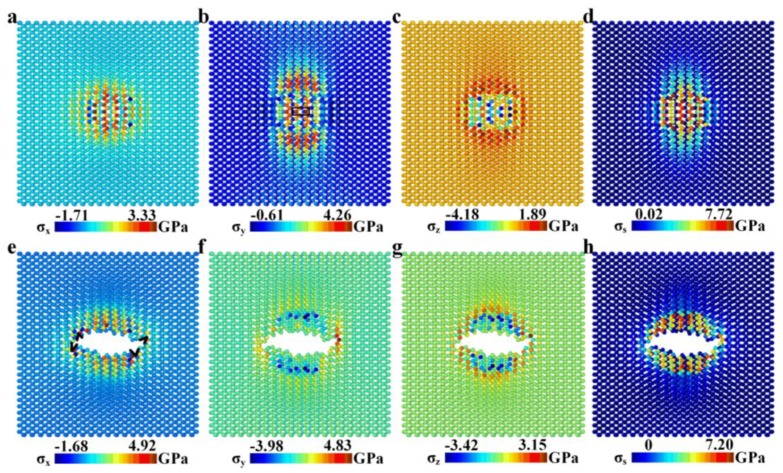
(**a**–**d**) Distribution of *σ*_x_, *σ*_y_, *σ*_z_ and *σ*_s_ in the freestanding BP monolayer before the generation of the first crack (corresponding to point A in Figure 2a); (**e**–**h**) Distribution of *σ*_x_, *σ*_y_, *σ*_z_ and *σ*_s_ in the freestanding BP monolayer before generation of the second crack (corresponding to point C in Figure 2a). The black sharp angles in Figure 5e represent the crack propagation tips.

For the indentation process of the freestanding BP monolayer, stress distributions before the first crack generated (corresponding to point A in Figure 2a, which is also the end of elastic deformation stage) and before the second crack started to grow (corresponding to point C in Figure 2a) were studied, as shown in Figure 5. 

Stress distribution in the freestanding BP monolayer at elastic stage is non-centrosymmetric, especially for *σ*_y_ and *σ*_s_, as shown in Figure 5a–d. The stressed range in zigzag direction is much larger than armchair direction, which is a result of the higher in-plane elastic modulus in zigzag direction of BP monolayer. The maximum normal stress component is 4.26 GPa for *σ*_y_ at the rectangle-boxed atoms in Figure 5b, where the first crack generates. 

As the first crack generates, on an integrated the freestanding BP monolayer, and the second crack generates on a cracked membrane, mechanisms for the generation and growth of the two cracks can be very different. Crack tips with atomic sharpness were found before the second crack started to grow, as shown in Figure 5e. While the first crack start from atoms of high tensile stress, the second crack start from the crack tips formed during the indentation process. The position and direction of the crack tips determine the growth behavior of the second crack.

**Figure 6:**
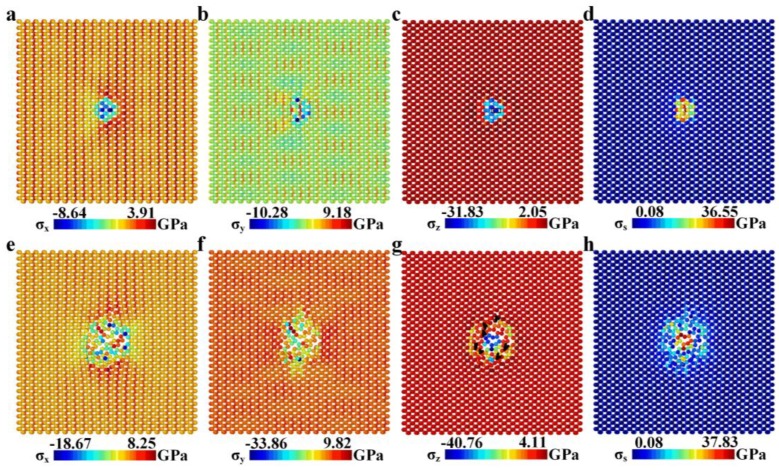
(**a**–**d**) Distribution of *σ*_x_, *σ*_y_, *σ*_z_ and *σ*_s_ in the Pt(111)-supported BP monolayer at the end of elastic deformation (corresponding to point A in Figure3a); (**e**–**h**) Distribution of *σ*_x_, *σ*_y_, *σ*_z_ and *σ*_s_ in the Pt(111)-supported BP monolayer at plastic deformation stage (corresponding to point C in Figure 3a). The black sharp angles in Figure 6g represent the crack propagation tips.

For the indentation process of the Pt(111)-supported BP monolayer, stress distribution at the end of elastic stage(corresponding to point A in Figure 3a) is more concentrated as shown in Figure 6, which is a result of the higher contact stiffness and smaller indentation depth. It can be found that the contact area between the diamond tip and the Pt-supported BP monolayer is elliptical, which is consistent with the theoretical model for anisotropic materials by Ciavarella and coworkers [51]. The maximum normal stress component is −31.83 GPa (the minus sign indicates the direction of stress) for *σ*_z_ at the rectangle-boxed atoms in Figure 6c, where plastic deformation appears. The maximum of *σ*_z_ far exceeds that of *σ*_x_ (−8.64GPa, see Figure 6a) and *σ*_y_ (−10.28GPa, see Figure 6b), which means the mainly deformation of the Pt(111)-supported BP monolayer at elastic stage is out-of-plane compression. The maximum of *σ*_z_ for the freestanding BP monolayer at the end of elastic stage is 4.18 GPa (see Figure 5c), far below that of the Pt(111)-supported BP monolayer, which means deformation of out-of-plane compression in the freestanding BP monolayer is far smaller than the Pt(111)-supported BP monolayer.

The fracture of the Pt(111)-supported BP monolayer mainly depends on the local stress state of out-of-plane compression. While the BP monolayer is remarkably anisotropic for in-plane mechanical properties, it is homogeneous for out-of-plane mechanical properties. Therefore, the fracture behavior for supported BP monolayer can be influenced greatly by the local morphology of the indenter. Unlike the freestanding BP monolayer, crack tips in the Pt(111)-supported BP monolayer at plastic deformation stage (corresponding to point C in Figure 3a) is unordered and asymmetric, as shown in Figure 6g. 

## 4. Conclusions

In conclusion, the fracture mechanism of BP monolayers under indentation was studied by molecular dynamics simulations. Fracture in a freestanding BP monolayer is firstly brittle-like and then ductile-like, with two cracks generating in order. Stress distribution and crack propagation in the freestanding BP monolayer show obvious directivity. Fracture in a Pt(111)-supported BP monolayer is ductile-like, with a plastic deformation stage following the elastic deformation stage. Stress distribution and crack propagation in the Pt(111)-supported BP monolayer do not show obvious directivity. While in-plane stretch is the main deformation for the freestanding BP monolayer, out-of-plane compression is the main deformation for the Pt(111)-supported BP monolayer. The difference between in-plane and out-of-plane mechanical properties leads to the different fracture behavior of the freestanding and Pt(111)-supported BP monolayers.

## Figures and Tables

**Table 1:** Parameters of Lennard Jones (LJ) potential used in this simulation.

Pair	C-P	C-Pt	Pt-P
*ε*_ij_ [meV]	6.878	38.635	91.209
*σ*_ij_ [Å]	3.4225	2.971	2.9565

## Supplementary Materials

The following are available online at http://www.mdpi.com/2079-4991/8/9/682/s1, Figure S1: The force-displacement (FN(d)) curve for the indentation process of the freestanding BP monolayer at different indentation speed; Figure S2: (**a**–**d**) side view of freestanding model during the indentation process, and Figure a, b, c, d correspond to point A, B, C, D in Figure 2a of the manuscript respectively. (**e**-**h**) cutaway view of Pt(111)-supported model during the indentation process, Figure e, f, g, h correspond to point A, B, C, D in Figure 3a of the manuscript respectively.
