# Supplementary material for: Atomic Scale Simulation on the Fracture Mechanism of Black Phosphorus Monolayer under Indentation
Source: Nanomaterials (Basel). 2018 Sep 1;8(9):682. doi: 10.3390/nano8090682 (PMC6163899; doi:10.3390/nano8090682)
Supplement: Supplementary file 1 [file nanomaterials-08-00682-s001.pdf]

Atomic Scale Simulation on the Fracture Mechanism of Black Phosphorus Monolayer under Indentation

Yang Liu, Yuhong Liu* and Jianbin Luo*

State Key Laboratory of Tribology, Tsinghua University, Beijing 100084, China; liuyang2013@gmail.com
* Correspondence: liuyuhong@tsinghua.edu.cn (Y.L.); luojb@tsinghua.edu.cn (J.L.); Tel.: +86-10-6278-8387 (Y.L.); +86-10-6278-1385 (J.L.)

In order to illustrate the influence of tip speed in this work, we conducted simulations at different tip speed, including 0.5 m/s and 10 m/s, as a supplement to the manuscript. The results are shown in the Figure s1. It can be found that tip speed has little influence on the results of indentation process when it is less than 10 m/s.

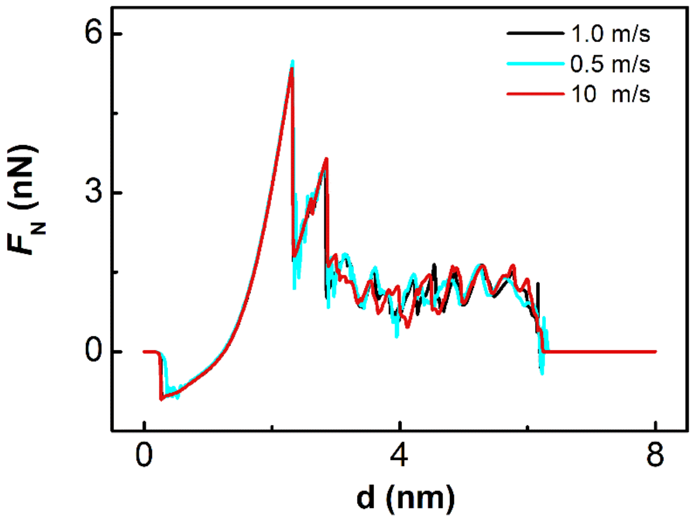

Figure S1. The force-displacement (FN(d)) curve for the indentation process of freestanding BP monolayer at different indentation speed.

In order to show the indentation process more clearly, the side view of freestanding model and cutaway view of Pt-supported model at different deformation stage are shown in Figure S2.

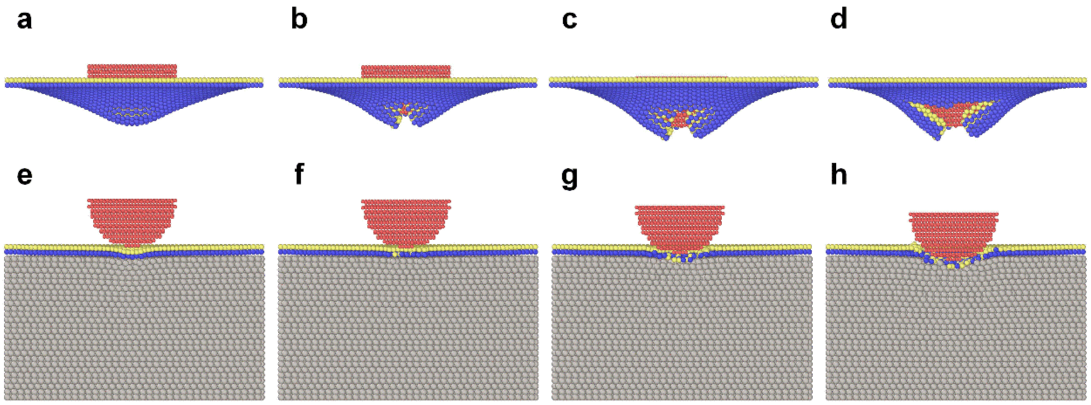

Figure S2. (a-d) side view of freestanding model during the indentation process, and Figure a, b, c, d correspond to point A, B, C, D in Figure 2a of the manuscript respectively. (e-h) cutaway view of Pt(111)-supported model during the indentation process, Figure e, f, g, h correspond to point A, B, C, D in Figure 3a of the manuscript respectively.
